# Pseudocannabinoid H4CBD improves glucose response during advanced metabolic syndrome in OLETF rats independent of increase in insulin signaling proteins

**DOI:** 10.1152/ajpregu.00125.2022

**Published:** 2023-10-30

**Authors:** Jessica N. Wilson, Dora A. Mendez, Francis Dhoro, Nikolay Shevchenko, Mark Mascal, Kyle Lund, Robert Fitzgerald, Nicholas V. DiPatrizio, Rudy M. Ortiz

**Affiliations:** ^1^Department of Molecular and Cell Biology, School of Natural Sciences, University of California, Merced, California, United States; ^2^Department of Chemistry, University of California, Davis, California, United States; ^3^Department of Pathology, University of California, San Diego, California, United States; ^4^Division of Biomedical Sciences, School of Medicine, University of California, Riverside, California, United States

**Keywords:** adiponectin, ghrelin, leptin, obesity, type II diabetes mellitus

## Abstract

Cannabidiol (CBD) use has grown exponentially more popular in the last two decades, particularly among older adults (>55 yr), though very little is known about the effects of CBD use during age-associated metabolic dysfunction. In addition, synthetic analogues of CBD have generated great interest because they can offer a chemically pure product, which is free of plant-associated contaminants. To assess the effects of a synthetic analogue of CBD (H4CBD) on advanced metabolic dysfunction, a cohort of 41-wk-old Otsuka Long-Evans Tokushima Fatty (OLETF) rats were administered 200 mg H4CBD/kg by oral gavage for 4 wk. Animals were fed ad libitum and monitored alongside vehicle-treated OLETF and Long-Evans Tokushima Otsuka (LETO) rats, the lean-strain controls. An oral glucose-tolerance test (oGTT) was performed after 4 wk of treatment. When compared with vehicle-treated, OLETF rats, H4CBD decreased body mass (BM) by 15%, which was attributed to a significant loss in abdominal fat. H4CBD reduced glucose response (AUC_glucose_) by 29% (*P* < 0.001) and insulin resistance index (IRI) by 25% (*P* < 0.05) compared with OLETF rats. However, H4CBD did not statically reduce fasting blood glucose or plasma insulin, despite compensatory increases in skeletal muscle native insulin receptor (IR) protein expression (54%; *P* < 0.05). H4CBD reduced circulating adiponectin (40%; *P* < 0.05) and leptin (47%; *P* < 0.05) and increased ghrelin (75%; *P* < 0.01) compared with OLETF. Taken together, a chronic, high dose of H4CBD may improve glucose response, independent of static changes in insulin signaling, and these effects are likely a benefit of the profound loss of visceral adiposity.

**NEW & NOTEWORTHY** Cannabis product use has grown in the last two decades despite the lack of research on Cannabidiol (CBD)-mediated effects on metabolism. Here, we provide seminal data on CBD effects during age-associated metabolic dysfunction. We gave 41-wk-old OLETF rats 200 mg H4CBD/kg by mouth for 4 wk and noted a high dose of H4CBD may improve glucose response, independent of static changes in insulin signaling, and these effects are likely a benefit of loss of visceral adiposity.

## INTRODUCTION

The popularity of cannabis use in older adults in the United States has more than doubled over the past two decades (1–4). Sentiments toward cannabis as a viable treatment intervention continue to be well documented as a low-risk alternative to pharmaceuticals (5, 6). Among the age-related ailments for which medicinal cannabis is sought, chronic pain, irritable bowel syndrome, and glaucoma are the most frequently cited (3, 4, 7). However, a common consequence of aging is impaired substrate metabolism, which can result in the onset of metabolic conditions ranging in severity from risk factors for metabolic syndrome (MetS) (8) to frank type II diabetes mellitus (T2DM; 9). The prevalence of MetS is 54.9% among people ≥60 yr of age (9) and the average age at diagnosis of T2DM is 46, with equal abundance across racial and ethnic groups (10). Importantly, the effects of herbal cannabis and cannabis constituent use during conditions of metabolic dysfunction are vastly understudied (11), and the explosion of interest in cannabis among older adults, coupled with the relatively high incidence of MetS in this population, constitutes a critical intersection between cannabis users and advanced metabolic dysfunction.

Cannabidiol (CBD) is an abundant, nonintoxicating constituent of *Cannabis sativa* that is of particular interest for pharmacological investigation. One drawback of CBD is, however, its easy conversion to tetrahydrocannabinol (THC), the psychotropic constituent of cannabis. This, and the legislative ambiguity and increasing ease of access to unregulated cannabis constituents, have prompted endeavors to synthetize analogues (‘pseudocannabinoids’) of the natural cannabinoids. The use of these synthetic analogues circumvents the complex regulatory landscape surrounding the production of scheduled compounds while at the same time providing a chemically homogeneous compound free from undesired phytocannabinoids or pesticides. Synthetic analogues of CBD can offer similar therapeutic effects to that of natural CBD. For example, dihydrocannabidiol (H2CBD) was found to reduce seizure frequency and severity in rats with equal effectiveness to that of CBD (12). Tetrahydrocannabidiol (H4CBD) is a compound that differs from CBD by the saturation of the two double bonds in the terpene fragment of the molecule. Like natural CBD, H4CBD has little affinity for the endocannabinoid receptors responsible for cannabis intoxication (13). Although the use of pseudocannabinoids in vivo has not been previously described in the context of metabolic dysfunction, in vitro use has yielded promising effects consistent with natural CBD (13–15). Therefore H4CBD, like H2CBD, is expected to exert similar effects to those of natural CBD.

Rigorous preclinical data are sparse concerning the effects of cannabinoids in models of metabolic dysfunction. Natural CBD reduced the incidence of diabetes in diet-induced obese (DIO) and nonobese diabetic mice (16, 17), providing some evidence that synthetic analogues likewise have the potential to similarly ameliorate dysregulated metabolism. Therefore, the goals of this study were to assess the effect of H4CBD on MetS cluster factors such as glucose intolerance and insulin resistance during advanced metabolic dysfunction using the Otsuka Long-Evans Tokushima Fatty (OLETF) rat as a monogenic model of diet-induced obesity that slowly manifests into insulin resistance, MetS, and ultimately frank T2DM (18, 19). At >40 wk of age, OLETF rats suffer from severe metabolic dysfunction (20) and therefore serve as a model of aged, acute MetS and T2DM common among older adults. We hypothesized that H4CBD would improve the glucose intolerance and insulin-resistant condition characteristic of this model.

## METHODS

All animal procedures were reviewed and approved by the institutional animal care and use committees of the University of California, Merced.

### Animals

Male lean Long Evan’s Tokushima Otsuka (LETO) rats and obese Otsuka Long Evans Tokushima Fatty (OLETF) rats (Japan SLC, Inc., Hamamatsu, Japan), both 14 wk of age, were assigned to the following groups (*n* = 8/group): *1*) vehicle-dosed LETO (LETO), *2*) vehicle-dosed OLETF (OLETF), and *3*) H4CBD-treated (200 mg/kg/day × 4 wk) OLETF (H4CBD). LETO rats were purposefully not treated, as the use of cannabinoids in healthy individuals is either preventative or recreational, neither of which addresses our hypothesis that cannabinoids will serve a therapeutic purpose during a condition of deranged metabolism. Rats were maintained in a specific pathogen-free, climate-controlled facility at the University of California Merced on a 12-h light:dark cycle (0700–1900 and 1900–0700, respectively). All animals had free access to water and were fed rat chow (Teklad Global; fat 9.0%; carbohydrate 44.9%; protein 19.0%) ad libitum. Treatment intervention was initiated at 41 wk of age. Phenotypic data [i.e., body mass (BM), food intake] were collected daily for all animals.

### Drug Preparation and Administration

H4CBD [1,2,8,9-tetrahydrocannabidiol, systematic name 2-(2-isopropyl-5-methylcyclohexyl)-5-pentylbenzene-1,3-diol], was prepared by reduction of synthetic H2CBD (8,9-tetrahydrocannabidiol) with hydrogen and a Pd/C catalyst in acetic acid solvent and was purified by distillation in vacuo. H2CBD was synthesized from olivetol and food-grade α-phellandrene according to the published procedure (12). All chemicals were purchased from Millipore Sigma and used as received.

Purified H4CBD (>99%) was suspended in food-grade sesame oil and administered by oral gavage at a dose of 200 mg/kg/day. A volumetrically equivalent dose of vehicle was administered to LETO and OLETF control groups by oral gavage. This dose, administered via intraperitoneal injection, has been shown to be similarly effective for the mitigation of seizure frequency and severity in rats compared with natural CBD (12), which gave reasonable cause for efficacy of H4CBD at the same dose, which is well below documented toxicity of CBD (>600 mg/kg) in rodents (21). Although this is a relatively high dose, the current study provides a basis to justify this dose as the highest needed.

### Oral Glucose Tolerance Test

At ∼45 wk of age and following an overnight fast, oral glucose-tolerance tests (oGTTs) were performed, as previously described (22). The positive incremental areas under the curve for glucose (AUC_glucose_) and insulin (AUC_insulin_) were calculated by the trapezoidal method (23). Insulin resistance index (IRI), an indirect marker of peripheral insulin action, was calculated by AUC_glucose_ × AUC_insulin_/100, as previously described (22, 24, 25). QUICKI was also calculated as 1/[log(I̥)+log(G̥)], as previously described (25, 26). Supplemental oGTT was performed as mentioned previously and intraperitoneal GTT utilized an identical glucose bolus dose injected into the intraperitoneal space.

### Tissue Collection

After the 4-wk study and 3 days following the oGTT, animals were fasted overnight and tissues were collected the following morning, as previously described (22, 27, 28). Briefly, trunk blood was collected into prepared, chilled glass tubes containing either heparin and protease inhibitor cocktail or EDTA, protease inhibitor cocktail, and angiotensin-converting enzyme inhibitor (22, 28, 29).

### Western Blot

Soleus was used to measure proteins involved in insulin signaling, as previously described (22, 25, 27, 30). Densitometry values were quantified using ImageJ software (NIH) and normalized by correcting for densitometry values of representative protein bands below 37 kDa stained with Ponceau S (31). Results are reported as expression (%) compared with LETO. Supplemental blots utilized β-actin (Cell Signaling Technology; 3700S) to normalize target protein signal in intestinal tissue.

### Drug Bioavailability Detection

H4CBD bioavailability in heparinized plasma was determined via HPLC and MS/MS, as described previously (32). In brief, 100 µL heparinized rat plasma was diluted with 100 µL Mass Spec Gold human serum (Golden West Diagnostics, MSG3200). The samples were analyzed using a WATERS TQS-Micro. The first quadrupole was set to 319.16 mass-to-charge ratio (*m*/*z*) and the third quadrupole isolated fragments of 181.02 *m*/*z*. Any sample that had a peak area count of <1,500 was considered negative for H4CBD.

### Plasma Analysis

Plasma concentrations of adiponectin (Millipore Sigma; EZRADP-62K), corticosterone (B; R&D Systems; KGE009), ghrelin (Sigma Aldrich; RAB0207), glucagon (Crystal Chemicals; 81519), and leptin (Millipore Sigma; EZRL-83K) were measured in fasted, end of study plasma samples. All samples were analyzed in duplicate and run in a single assay with intra-assay and percent CV of <10% for all assays.

### Statistics

All values are represented as means ± SE unless otherwise indicated. Means were compared by one-way ANOVA followed by Tukey’s honestly significant difference or unpaired, one-tailed *t* test to assess significant differences among groups. Means and regressions were considered significant at *P* < 0.05. Outliers were detected by ROUT (*Q* = 1.0%) and removed; however, it should be noted that this was necessary for only 12 occurrences accounting for <1% of data presented here. All statistical procedures, including Pearson *r* correlations, were performed using GraphPad Prism 9.3.1 (GraphPad Software, Inc., San Diego, CA).

## RESULTS

### H4CBD Was Only Detected in Plasma from Treated Rats

**Figure 1.**
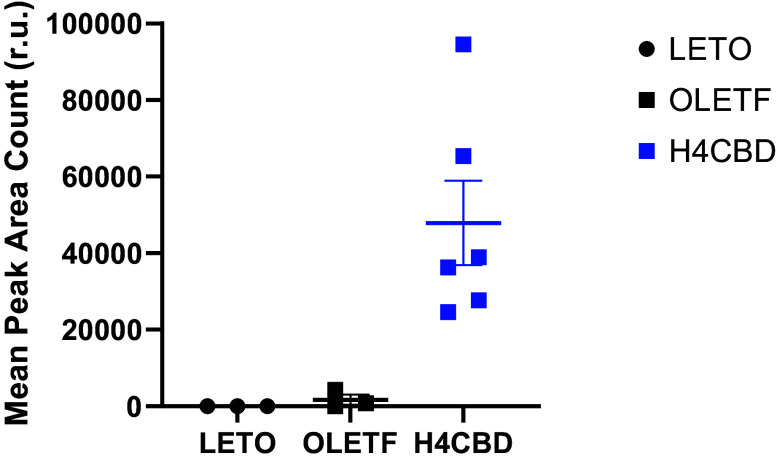
Bioavailability of drug in end of study plasma. Means ± SE. Plasma AUC relative abundance of H4CBD in 45-wk-old LETO (*n* = 3), OLETF (*n* = 3), and H4CBD-treated OLETF (H4CBD; *n* = 6). LETO, long Evan’s Tokushima Otsuka; OLETF, Otsuka Long-Evans Tokushima Fatty.

Bioavailability of H4CBD compound was validated in the end of study plasma and confirmed that only treated animals received drug (Fig. 1). Because of the novelty of the compound, any sample that had a peak area count of <1,500 was considered negative for H4CBD.

### H4CBD Reduced BM Independent of Changes in Food Consumption

BM and food consumption were measured daily, and abdominal fat masses were weighed at dissection to determine the effect of H4CBD treatment on phenotypic indicators of metabolic dysfunction. H4CBD reduced BM to LETO levels (Table 1). BM of OLETF was 16% higher than LETO (*P* < 0.05) and 15% higher (*P* < 0.05) than the H4CBD group (Table 1). Relative retroperitoneal fat, but not relative epidydimal fat, was 267% more abundant in OLETF than LETO (*P* < 0.0001; Table 1). H4CBD reduced relative retroperitoneal fat by 24% (n.s.) and relative epidydimal fat by 35% (*P* < 0.05) compared with OLETF (Table 1). Relative visceral (epi + retro) adipose was 149% higher in OLETF compared with LETO (*P* < 0.0001), which tended to reduce by 25% with H4CBD treatment (*P* = 0.057; Table 1). Initial and final food intake between OLETF and H4CBD were essentially identical (Table 1).

**Table 1.** Morphometrical measurements in LETO, OLETF, and H4CBD-treated OLETF male rats

	LETO	OLETF	H4CBD
Body mass (g) first day of study	534 ± 7	634 ± 31*	598 ± 27*
Body mass (g) last day of study	527 ± 10	640 ± 38*	516 ± 21
Relative retro. fat, g/100 g BM	2.4 ± 0.1	8.6 ± 0.6*	6.6 ± 0.9*
Relative epi. fat, g/100 g BM	1.8 ± 0.1	1.8 ± 0.2	1.2 ± 0.1*
Relative visceral fat, g/100 g BM	4.2 ± 0.2	10.5 ± 0.7*	7.8 ± 1.1*
Initial, absolute food intake, g	25 ± 2	32 ± 1*	34 ± 2*
Final, absolute food intake, g	21 ± 1	37 ± 2*	36 ± 2*
Relative final food intake, g/100 g BM	3.9 ± 0.1	6.1 ± 0.7*	6.7 ± 0.4*

Values are mean ± SE. BM, body mass; epi., epidydimal; HSD, honestly significant difference; LETO, long Evan’s Tokushima Otsuka; OLETF, Otsuka Long-Evans Tokushima Fatty; retro., retroperitoneal, visceral, epi + retro, initial food intake (average of 3 days immediately before dosing period) final food intake (average of last 3 days of dosing period). *n* = 8/group. *P < 0.05 difference from LETO, *P* < 0.05 difference from OLETF by one-way ANOVA with Tukey’s HSD or unpaired, one-tailed *t* test.

### H4CBD Ameliorated Dynamic Glucose Response but Did Not Reverse Diabetic Phenotype

**Figure 2.**
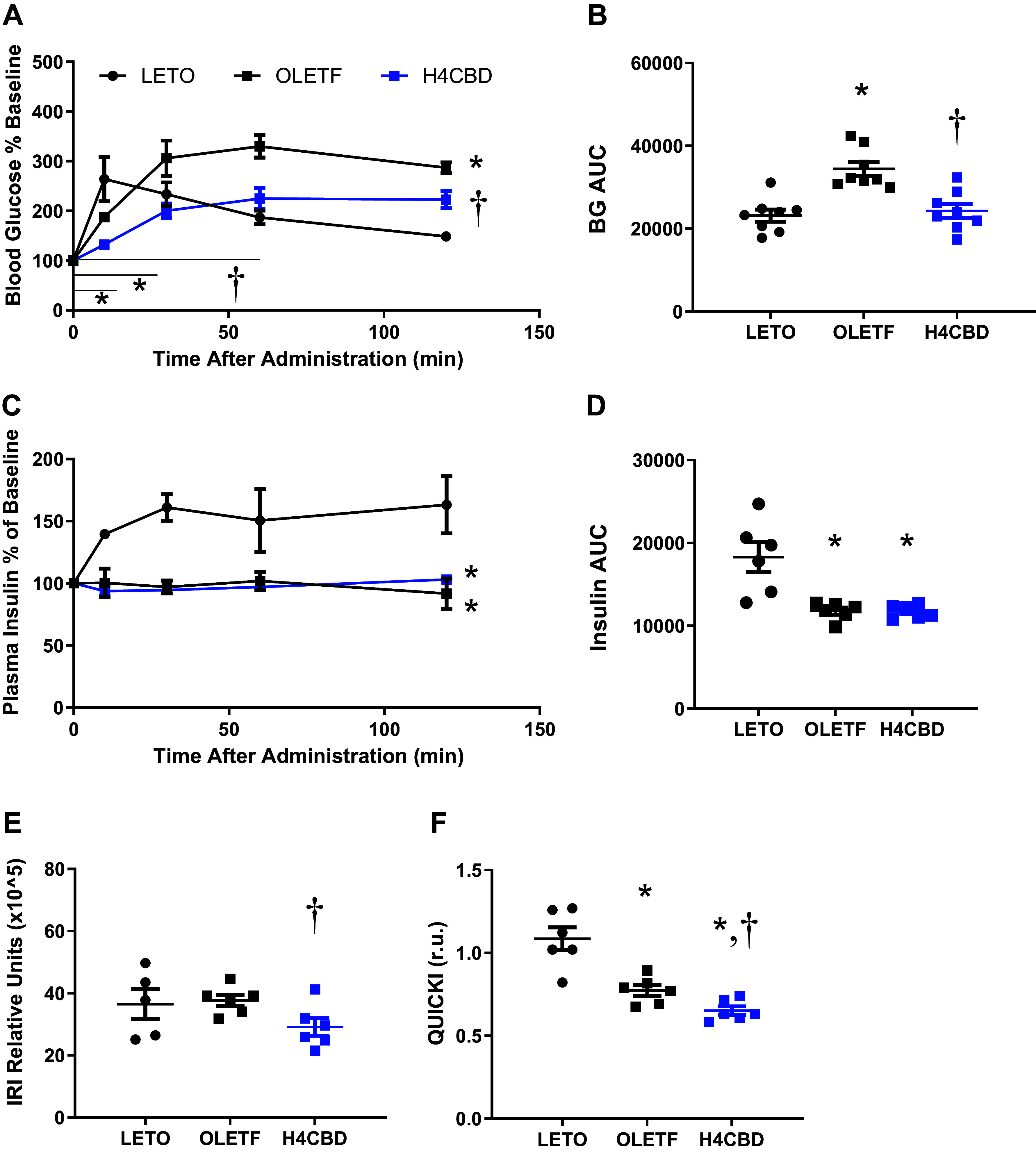
H4CBD ameliorated glucose response but not insulin resistance and sensitivity indices in advanced MetS. Mean ± SE. Blood glucose % baseline response to oGTT (*A*) and corresponding AUC (r.u.) (*B*), insulin response % baseline to oGTT (*C*) and corresponding AUC (r.u.) (*D*), insulin resistance index (IRI; relative units) (*E*) and QUICKI in 45-wk-old LETO (*n* = 8), OLETF (*n* = 8), and H4CBD-treated OLETF (H4CBD; *n* = 8) (*F*). *P < 0.05 difference from LETO and †*P* < 0.05 difference from OLETF by one-way or two-way ANOVA with Tukey’s HSD or by unpaired, one-tailed *t* test. HSD, honestly significant difference; LETO, long Evan’s Tokushima Otsuka; oGTT, oral glucose-tolerance test; OLETF, Otsuka Long-Evans Tokushima Fatty.

oGTTs were performed, wherein plasma insulin was measured at corresponding timepoints, to determine the effects of H4CBD on glucose intolerance. From these measurements, insulin resistance index (IRI) and QUICKI were calculated to determine the effect of H4CBD on peripheral insulin sensitivity during advanced MetS. LETO blood glucose response peaked at 10 min after glucose bolus, whereas OLETF peaked 60 min after glucose bolus (Fig. 2*A*). AUC_glucose_ of discrete time points (T0-T60) were 32% lower for H4CBD compared with OLETF (*P* < 0.01) suggesting a delay of glucose absorption into the bloodstream (Fig. 2*A*). Glucose response curve was lowest for H4CBD-treated animals overall (*P* < 0.01; Fig. 2*A*). Overall AUC_glucose_ was 49% higher in OLETF compared with LETO (*P* < 0.001) and H4CBD reduced AUC_glucose_ 29% from OLETF (*P* < 0.001; Fig. 2*B*). Plasma insulin response was abolished in OLETF, which was not rescued by H4CBD treatment (Fig. 2, *C* and *D*). The calculated insulin resistance index (IRI) status was similar between aged LETO and OLETF rats, but IRI was reduced by 23% in H4CBD compared with OLETF (*P* < 0.001; Fig. 2*E*). H4CBD reduced QUICKI by 16% from OLETF (*P* < 0.01), providing an additional measure of reduced insulin resistance (Fig. 2*F*).

### H4CBD Did Not Modulate Gut-Mediated Glucose Uptake

Upon comparison of oral versus intraperitoneal GTTs in a small cohort, it was determined that H4CBD did not induce a functional reduction in gut-mediated glucose uptake (Supplemental Fig. S1, *A–D*). Moreover, intestinal GLUT4 expression was not significantly altered by H4CBD (Supplemental Fig. S2, *A–C*).

### H4CBD Did Not Improve Static Indicators of Glucose Tolerance

At 45 wk of age, fasting blood glucose (FBG) was 44% (*P* < 0.05) higher in OLETF than LETO whereas levels were 2.2-fold higher in H4CBD than LETO (*P* < 0.0001) and 56% higher than OLETF (*P* < 0.01; Table 2). Fasting plasma insulin was 64% higher in OLETF compared with LETO (*P* < 0.01) but similar to H4CBD (Table 2). Plasma glucagon was 63% lower in OLETF compared with LETO (*P* < 0.05) but was not different from H4CBD (Table 2). H4CBD increased fasting glucose-to-insulin ratio by 47% (*P* < 0.05) and reduced fasting insulin to glucagon ratio by 64% (*P* < 0.05) compared with OLETF (Table 2).

**Table 2.** End of study fasting plasma measurements

Strain	LETO	OLETF	H4CBD
Fasting blood glucose, mmol/L	7.0 ± 0.4	10 ± 1.0*	16 ± 1.9*
Fasting insulin, μIU/mL	7.7 ± 0.7	13 ± 0.9*	14 ± 0.4*
Fasting glucagon, pg/mL	22 ± 5.0	8.0 ± 1.3*	9.4 ± 9.4*
Glucose:Insulin, mmol/μIU × 104	8.7 ± 0.6	7.4 ± 0.7	11 ± 1.1
Insulin:Glucagon, μIU/pg	0.4 ± 0.0	1.6 ± 0.1*	0.6 ± 0.0

Values are mean ± SE; *n* = 5–8/group. HSD, honestly significant difference; LETO, long Evan’s Tokushima Otsuka; OLETF, Otsuka Long-Evans Tokushima Fatty. *P < 0.05 difference from LETO, *P* < 0.05 difference from OLETF by one-way ANOVA with Tukey’s HSD or unpaired, one-tailed *t* test.

### H4CBD Statically Increased Insulin Receptor Expression

**Figure 3.**
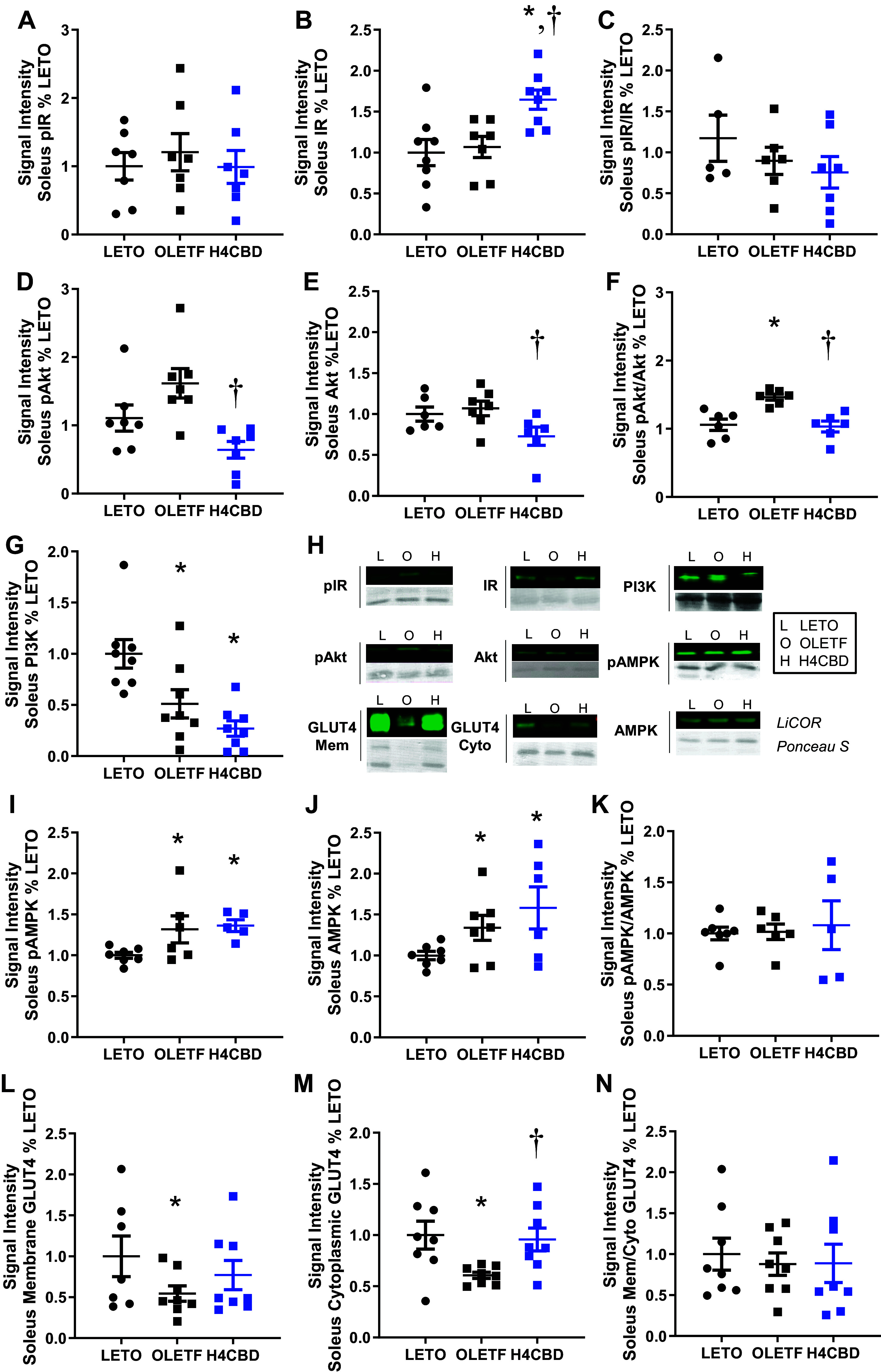
H4CBD induced compensatory increase in skeletal muscle insulin receptor (IR) expression. Means ±SE. pIR (*A*), IR (*B*), pIR/IR (*C*), pAkt (*D*), Akt (*E*), pAkt/Akt (*F*), PI3K (*G*), representative blots (*H*), pAMPK *(I*), AMPK (*J*), pAMPK/AMPK (*K*), membrane-bound GLUT4 (*L*), cytoplasmic GLUT4 (*M*), and Mem/cyto GLUT4 soleus protein expression (*N*) in 41–45-wk-old LETO (*n* = 8), OLETF (*n* = 8), and H4CBD-treated OLETF (H4CBD; *n* = 8). *P < 0.05 difference from LETO and †*P* < 0.05 difference from OLETF by one-way ANOVA with Tukey’s HSD or by unpaired, one-tailed *t* test. HSD, honestly significant difference; LETO, long Evan’s Tokushima Otsuka; OLETF, Otsuka Long-Evans Tokushima Fatty.

Because skeletal muscle is the primary sink for glucose utilization, insulin signaling proteins were measured to determine effects to static tone of proteins in the insulin signaling cascade. No changes were detected in the abundance of static, phosphorylated insulin receptor (pIR) (Fig. 3*A*). H4CBD increased native insulin receptor (IR) expression by 54% over OLETF (*P* < 0.05) (Fig. 3*B*) but pIR/IR was not changed (Fig. 3*C*). Cytosolic pAkt and Akt expressions were comparable between LETO and OLETF but H4CBD reduced the expressions of pAkt, Akt, and pAkt:Akt ratio by 58% (*P* < 0.05), 32% (*P* < 0.05), and 35% (*P* < 0.01), respectively, compared with OLETF (Fig. 3, *D–F*). PI3K expression was reduced by 49% (*P* < 0.05) in OLETF compared with LETO, and H4CBD tended (*P* = 0.07) to further reduce PI3K expression by 47% compared with OLETF (Fig. 3*G*). pAMPK was 34% (*P* < 0.05) higher in OLETF compared with LETO, and H4CBD had no effect (Fig. 3*I*). Native AMPK was 32% (*P* < 0.05) higher in OLETF compared with LETO but H4CBD had no effect (Fig. 3*J*). No changes were detected in the ratio of pAMPK/AMPK expression (Fig. 3*K*).

Translocated GLUT4 may serve as a static indicator of increased insulin signaling and was measured by probing for its expression in the membrane fraction of skeletal muscle. Translocated GLUT4 was 46% (*P* < 0.05) lower in OLETF compared with LETO, which H4CBD did not rescue (Fig. 3*L*). Cytosolic GLUT4 was 39% lower in OLETF compared with LETO (*P* < 0.05) and 58% (*P* < 0.01) higher in H4CBD compared with OLETF (Fig. 3*M*). The ratio of membrane to cytosolic GLUT4 was unchanged among the groups (Fig. 3*N*).

### H4CBD Reduced Circulating Adiponectin and Leptin and Increased Ghrelin

**Figure 4.**
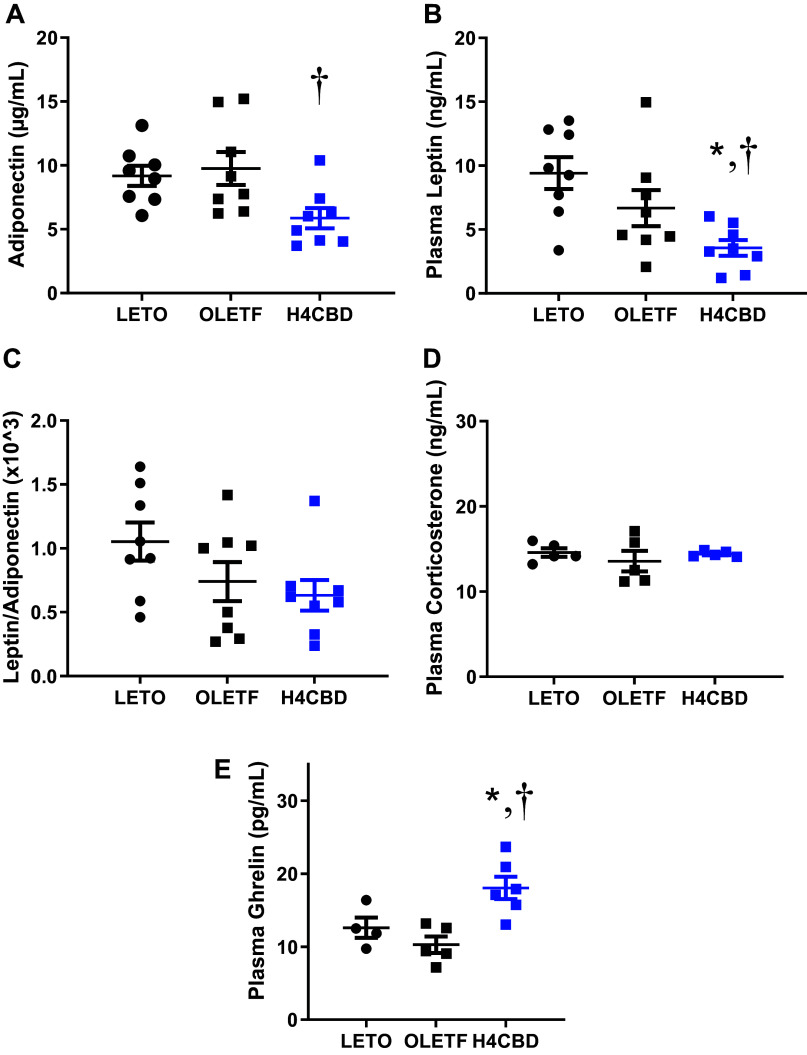
H4CBD reduced circulating adipokines but increased hunger hormone ghrelin in advanced MetS. Means ± SE. Plasma adiponectin (*A*), plasma leptin (*B*), plasma leptin:adiponectin (*C*), plasma corticosterone (*D*), and plasma ghrelin (*E*) in 45-wk-old LETO (*n* = 8), OLETF (*n* = 8), and H4CBD-treated OLETF (H4CBD; *n* = 8). *P < 0.05 difference from LETO and †*P* < 0.05 difference from OLETF by one-way ANOVA with Tukey’s HSD or by unpaired, one-tailed *t* test. HSD, honestly significant difference; LETO, long Evan’s Tokushima Otsuka; OLETF, Otsuka Long-Evans Tokushima Fatty.

Fasting plasma adiponectin, corticosterone, ghrelin, and leptin were measured to assess the effect of H4CBD on levels of adipocytokines and other hormones associated with insulin resistance and obesity. Although there was no strain difference observed, H4CBD reduced circulating adiponectin by 40% (*P* < 0.05) compared with OLETF (Fig. 4*A*). Leptin tended to be reduced in OLETF compared with LETO (*P* = 0.08) and H4CBD reduced leptin by 47% (*P* < 0.05) compared with OLETF (Fig. 4*B*). The leptin-to-adiponectin ratio was comparable among groups (Fig. 4*C*) along with plasma corticosterone (Fig. 4*D*). Plasma ghrelin was 75% (*P* < 0.05) greater in H4CBD compared with the other groups, and levels were similar between LETO and OLETF (Fig. 4*E*).

### H4CBD-Induced Reduction of Abdominal Fat Was Positively Correlated With Adiponectin Reduction

**Figure 5.**
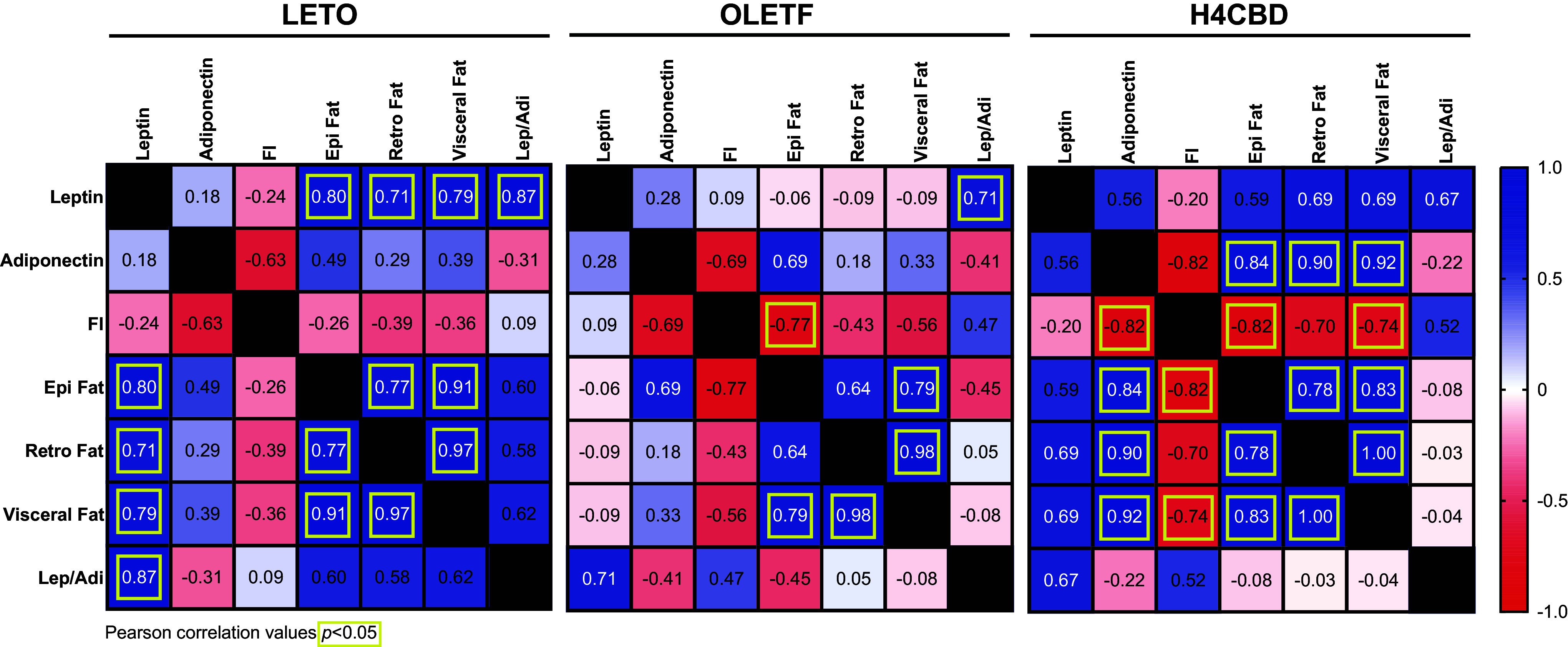
H4CBD-induced reductions of abdominal fat and plasma adiponectin are positively correlated. Pearson *r* correlation values of end of study leptin, adiponectin, food intake (FI), epidydimal (Epi) fat, retroperitoneal (Retro) fat, visceral (Epi + Retro) fat, leptin:adiponectin in LETO (*n* = 8), OLETF (*n* = 8), and H4CBD (*n* = 8). LETO, long Evan’s Tokushima Otsuka; OLETF, Otsuka Long-Evans Tokushima Fatty.

Pearson *r* correlations of end-of-study plasma leptin, adiponectin, food intake, epidydimal fat, retroperitoneal fat, visceral (combined) fat, and leptin-to-adiponectin ratio were conducted to determine significant interactions between fat mass and hormones associated with insulin resistance and obesity. LETO visceral fat was positively associated with plasma leptin (Pearson *r* 0.79; *P* < 0.05) unlike OLETF (Fig. 5). The H4CBD-induced reduction in visceral fat was positively correlated with plasma adiponectin (Pearson *r* 0.92; *P* < 0.01) and negatively correlated with food intake (Pearson *r* −0.74; *P* < 0.05) (Fig. 5).

## DISCUSSION

As the prevalence of MetS continues to increase in the United States and globally, identifying novel compounds to ameliorate the multiple morbidities that characterize MetS becomes increasingly important. Addressing the many maladies that comprise MetS simultaneously is challenging, which contributes to the urgency to develop novel therapies. A review of the potential benefits of cannabinoids on a wide range of pathologies is encouraging; however, the effects on the various pathologies constituting MetS are largely unknown. A few studies have shown beneficial (33, 34) or null effects (35) of CBD on isolated MetS risk factors, but these effects have not been explored in context of cluster factors. Therefore, the aim of this study was to assess the effects of a closely analogous pseudocannabinoid on glucose intolerance in the condition of advanced MetS, which is most frequently observed in older populations, while averting the use of a herbal cannabinoid. We chose for this purpose to evaluate the effects of the synthetic, non-narcotic cannabinoid analogue 1,2,8,9-tetrahydrocannabidiol (H4CBD) because of its synthetic accessibility in pure form and potential for wider adoption than CBD, which is subject to multiple regulatory restrictions worldwide.

A hallmark of MetS is sustained hyperglycemia, with or without insulin resistance, whereby blood glucose levels remain elevated. OLETF rats fed the same diet as their lean, strain-control, LETO rats develop later onset hyperglycemia by 15 wk of age (27), which has been shown to be attenuated by caloric restriction at earlier stages of disease progression (22, 36). Although the primary etiologic event for hyperinsulinemia is not explicit, OLETF demonstrates pancreatic β-cell dysfunction by 16 wk marked by insulin resistance and impaired insulin secretion (20). We show that in older OLETF rats (>40 wk of age) fed ad libitum, fasting blood glucose (FBG) was 43% higher than LETO and that OLETF rats struggled to clear glucose from circulation long after time-to-clearance in LETO. Both metrics are consistent with previously characterized hyperglycemia in OLETF rats at 40 wk of age (20). Moreover, the insulin response to oGTT was severely blunted at this age, though fasting insulin remained relatively high in response to sustained hyperglycemia, which is indicative of *1*) insufficient β-cell insulin secretion (37) and *2*) insulin resistance at the level of the receptor (38), respectively. Most importantly, treatment with H4CBD mitigated the dynamic, strain-associated glucose intolerance independent of static enhancements of proteins of the insulin signaling pathway.

Endocannabinoid signaling in the gut and peripherally has been shown to modulate nutrient absorption and uptake (39, 40), which could confer a beneficial delay in the uptake of glucose into circulation. Thus, the improvement in glucose tolerance observed with H4CBD may have been accomplished via delayed absorption of glucose across the gut independent of static changes in insulin signaling. However, a separate study, which used small cohort (*n* = 5/group) of younger animals, did not support this argument (Supplemental Fig. S1). Nor was a difference in intestinal GLUT4 expression measured (Supplemental Fig. S2). That said, because glucose-stimulated insulin secretion was blunted in both older OLETF groups, the reduction in the IRI calculation in the H4CBD group was likely more of a function of reduced AUC_glucose_ than insulin-dependent mechanisms. The reduction in visceral adiposity may have also contributed to the improvement in glucose tolerance. Regardless of the primary mechanisms, the result of 4 wk of H4CBD treatment was an improvement in glucose tolerance subsequent to the insulin resistance status.

Interestingly, although H4CBD resulted in acute, dynamic improvements in glucose metabolism, the treatment did not appear to correct the chronically sustained hyperglycemia or hyperinsulinemia, suggesting that this dose and duration of treatment were not sufficient to reverse the diabetic phenotype.

Skeletal muscle is considered the primary sink for glucose metabolism, which is predominantly regulated by insulin receptor-mediated signaling (41). At the cellular level, IR expression in skeletal muscle of H4CBD rats was higher than the other groups, although the phosphorylation of IR was not different, resulting in a reduced ratio and suggesting that IR activation was suppressed or at least not chronically and statically changed. The increase in native IR protein expression may be a compensatory response to the static hyperglycemia and hyperinsulinemia in this group, although humans and mice with insulin resistance have reduced IR expression (42) and the treatment of hyperglycemia with berberine (a natural alkaloid) has been shown to increase IR expression (43). Alternatively, improved protein stability (reduced susceptibility to degradation) could contribute to the observed hyperinsulinemia and enhanced IR protein expression (44). Regardless, the inherent limitation of this interpretation is the single time point at which these measurements were made. Similar analyses at various timepoints would reinforce this argument to interpret the direct or indirect dynamic effects of H4CBD on the insulin signaling cascade in skeletal muscle.

The loss of visceral adipose invariably affects circulating adipokines, which influence peripheral insulin resistance and modulate hunger (45, 46). The decreases in the visceral adipose depots in response to H4CBD treatment translated into reductions in plasma adiponectin and leptin and likely reflect the inability to restore hormone-driven insulin sensitization in skeletal muscle. Although increased leptin may impair insulin sensitivity and adiponectin can counteract leptin to improve sensitivity, the simultaneous reductions in both likely contributed to the apparent insulin resistance of skeletal muscle in this model. In addition, the reductions in both adipokines and their ratios suggest that H4CBD treatment may have shifted substrate metabolism, resulting in increased lipid catabolism and ultimately reduced adiposity. Preferential enhancement in lipid catabolism may compensate for the energy deficit conferred by insulin resistance but it is not clear if this compensation is sustainable or whether these effects would be lost after cessation of treatment and warrants further investigation.

Though not statistically correlated, the increase in plasma ghrelin supports the observed increase in relative food intake in the presence of body mass loss. However, it is unclear if H4CBD directly stimulated ghrelin secretion or if it was compensatory in response to the loss in fat mass, and thus, responding to a shift in substrate metabolism to help maintain energetic homeostasis (47–49).

### Perspectives and Significance

In summary, the H4CBD-mediated nuanced improvement in dynamic glucose tolerance observed here was biologically significant and likely attributed, at least in part, to the reduction in adiposity and/or modulation of nutrient absorption by the gut. Although the improvement was modest, it is important to note that the conditions under which these benefits were realized are highly dysfunctional. The animals in this study were advanced in age with severe metabolic derangement, rendering any health benefits at this stage is remarkable. It is likely that intervention at an earlier stage of disease progression could have a more profound or potentially preventative effect on MetS cluster factors. Future preclinical research should determine not only the ideal timepoint to initiate a therapeutic intervention but minimum dose to effect and ideal dose duration as well as the efficacy of intermittent dosing. Because both natural phytocannabinoids and synthetic pseudocannabinoids are tolerated similarly (12, 50), there exists potential for similar therapeutic efficacy in other systems as they relate to amelioration of metabolic dysfunction. The potential benefits on ameliorating glucose intolerance during advanced MetS observed here are encouraging and contribute to establishing a foundation from which to inform future studies on the effects of CBD, H4CBD, and other synthetic CBD analogues on metabolic disorders.

## DATA AVAILABILITY

The datasets generated for this study are available upon request to the corresponding author.

## SUPPLEMENTAL DATA

10.6084/m9.figshare.20018018 Supplemental Figs. S1 and S2: https://doi.org/10.6084/m9.figshare.20018018.

## GRANTS

J.N.W. and some of the analyses were supported by CMCR pilot Grant A21-0086 awarded to R.M.O.

## DISCLOSURES

No conflicts of interest, financial or otherwise, are declared by the authors.

## AUTHOR CONTRIBUTIONS

J.N.W., M.M., N.V.D., and R.M.O. conceived and designed research; J.N.W., D.A.M., F.D., and K.L. performed experiments; J.N.W., K.L., R.F., and R.M.O. analyzed data; J.N.W. and R.M.O. interpreted results of experiments; J.N.W. prepared figures; J.N.W. drafted manuscript; J.N.W., N.S., M.M., K.L., R.F., N.V.D., and R.M.O. edited and revised manuscript; J.N.W., D.A.M., F.D., N.S., M.M., K.L., R.F., N.V.D., and R.M.O. approved final version of manuscript.
